# Comparative Analysis of Cervical Spine Management in a Subset of Severe Traumatic Brain Injury Cases Using Computer Simulation

**DOI:** 10.1371/journal.pone.0019177

**Published:** 2011-04-22

**Authors:** Kimbroe J. Carter, C. Michael Dunham, Frank Castro, Barbara Erickson

**Affiliations:** 1 Medical Decision Making Society of Youngstown Ohio, St. Elizabeth Health Center, Youngstown, Ohio, United States of America; 2 Trauma/Critical Services, St. Elizabeth Health Center, Youngstown, Ohio, United States of America; 3 Department of Pathology, Northeastern Ohio Universities Colleges of Medicine and Pharmacy, Rootstown, Ohio, United States of America; Virginia Commonwealth University Rehabilitation and Research Center, United States of America

## Abstract

**Background:**

No randomized control trial to date has studied the use of cervical spine management strategies in cases of severe traumatic brain injury (TBI) at risk for cervical spine instability solely due to damaged ligaments. A computer algorithm is used to decide between four cervical spine management strategies. A model assumption is that the emergency room evaluation shows no spinal deficit and a computerized tomogram of the cervical spine excludes the possibility of fracture of cervical vertebrae. The study's goal is to determine cervical spine management strategies that maximize brain injury functional survival while minimizing quadriplegia.

**Methods/Findings:**

The severity of TBI is categorized as unstable, high risk and stable based on intracranial hypertension, hypoxemia, hypotension, early ventilator associated pneumonia, admission Glasgow Coma Scale (GCS) and age. Complications resulting from cervical spine management are simulated using three decision trees. Each case starts with an amount of primary and secondary brain injury and ends as a functional survivor, severely brain injured, quadriplegic or dead. Cervical spine instability is studied with one-way and two-way sensitivity analyses providing rankings of cervical spine management strategies for probabilities of management complications based on QALYs. Early collar removal received more QALYs than the alternative strategies in most arrangements of these comparisons. A limitation of the model is the absence of testing against an independent data set.

**Conclusions:**

When clinical logic and components of cervical spine management are systematically altered, changes that improve health outcomes are identified. In the absence of controlled clinical studies, the results of this comparative computer assessment show that early collar removal is preferred over a wide range of realistic inputs for this subset of traumatic brain injury. Future research is needed on identifying factors in projecting awakening from coma and the role of delirium in these cases.

## Introduction

Of the 275,000 individuals in the United States hospitalized each year for traumatic brain injury (TBI) a small number, less than 1 percent of those hospitalized, result in severe brain injury with cervical spine instability which is undetected by both computerized tomography and neurologic exam for motor deficits in the emergency room. This injury, caused by damaged ligaments, predisposes them to quadriplegia [1], [2]. In cases of TBI, paramedics use a cervical collar in order to stabilize the neck should cervical spine instability be present. However, in caring for severe TBI with possible cervical spine instability, there is the concern that use of cervical collars and cervical spine magnetic resonance imaging (MRI) intended to avoid quadriplegia are associated with complications which may contribute to additional brain injury [2]. These complications include increased intracranial pressure, delirium, and ventilator associated pneumonia (VAP).

The cervical collar may increase intracranial pressure by impeding jugular venous return and as a physical restraint may be associated with increased delirium. Agitation, increased intrathoracic pressure and associated intracranial pressure may contribute to worsening of TBI outcomes. Recumbency for the MRI predisposes to aspiration which acutely can lead to hypoxemia and subsequent secondary brain injury as well as develop into VAP.

Consequences of management decisions have lifetime affects. No controlled clinical trial has addressed the long term merits of management approaches using cervical collar and cervical spine MRI in the group of severely brain injured that have a negative cervical spine computerized tomogram and no motor deficits in emergency room. This group includes individuals with undetected cervical ligament instability at risk for quadriplegia. In the absence of clinical trial data, there are divergent opinions as to the care of these individuals. The purpose of this study is to compare health outcomes of the four cervical spine management strategies in treating this group.

This study uses a computer model to measure the quality adjusted life years (QALYs) resulting from cervical spine management in cases with severe TBI who are at risk for cervical spine instability solely due to damaged ligaments. Cervical spine instability and vertebral fracture is common but the condition of instability due solely to ligament damage is infrequent [3]. Application of a cervical collar benefits only those uncommon cases with ligament damage and no vertebral fracture. Prolonged cervical collar use poses a risk of additional brain injury and worsening outcomes. The model evaluates four cervical spine management strategies after neck evaluation with no apparent spinal deficit while in the emergency room and a negative computerized tomography of the cervical spine excluding the possibility of fractures of cervical vertebrae.

In building the decision tree a given strategy is measured by combining applicable chance events, outcomes and contributions to brain injury into clinically possible relations. These arrangements are programmed in a left to right manner with branching to represent the dependency of intermediate outcomes. This process simulates early and late complications that an individual might experience. The pathways have a tree-like appearance and each decision tree has approximately 40 branches. Health outcomes are the terminal branches.

This model simulates the degree of primary and secondary brain injury resulting from the trauma, the possibility of quadriplegia from cervical spine instability and the consequences of brain injury resulting from the complications of cervical spine management. The four heath outcomes considered are functional survival, severely brained injured, quadriplegic or death. Functional survivals are capable of independent activities of daily living, while cases with severe brain disability are not and quadriplegics have total loss of use of all four limbs. The accumulation of health outcomes for the cases permits the evaluation of the four cervical spine management strategies.

## Methods

### Model Overview

Cases of severe TBI with possible cervical spine instability are modeled with different survival and health outcomes determined by the severity of brain injury and cervical spine management complications occurring over the duration of therapy. Ideas for the model come from a literature report which describes these injuries as high risk, unstable and stable with estimates of functional survival from the trauma as 150, 350 and 600 per 1000 cases respectively [2]. The scheme of classification of TBI used in this model is based on the Glasgow Coma Scale, age, and the presence or absence of increased intracranial pressure, early ventilator associated pneumonia, hypoxemia and hypotension [2]. Cases in the unstable category have intracranial hypertension, hypoxemia, hypotension, or early ventilator associated pneumonia. Those cases in the high risk category have an admission GCS 3–5 or are greater than 45 years of age without intracranial hypertension, hypoxemia, hypotension, or early VAP. Cases in the stable category have an admission GCS 6–8 and are between the ages of 15 and 45 years without intracranial hypertension, hypoxemia, hypotension, or early VAP.

Cervical spine instability may occur with trauma and severe brain injury as a result of isolated ligament damage [4], [5]. Cervical spine instability predisposes a case to quadriplegia. The probabilities of events that contribute to brain injury and the development of quadriplegia, as well as the life expectancy associated with health outcomes have been estimated from literature review or expert opinion and are used as inputs in the model [6].

To reduce the risk of quadriplegia, severe TBI cases may be managed with a cervical collar or a cervical spine MRI evaluation. Treatments are adjusted according to the results of the imaging [2], [7]. However, use of a cervical collar and the MRI carry inherent risks of complications which may increase brain injury.

The management strategies for severe TBI include 4 cervical spine protocols. Considered strategies are early collar removal occurring by day three (ECR), late collar removal following day 14 (LCR), early collar removal with MRI (ECR/MRI) and late collar removal with MRI (LCR/MRI). The duration that the cervical collar is worn increases the risk of intracranial hypertension and delirium resulting from the restraint imposed by the collar [8]. The transport from the intensive care unit and supine positioning needed for cervical spine MRI increases the risk of intracranial hypertension and aspiration resulting in ventilator associated pneumonia [2], [9]–[12].

### Decision Nodes and Chance Events

Each type of TBI, unstable, high risk and stable, is modeled as a separate decision tree using the software application TreeAge Pro Suite 2008 in cohorts of 100,000. The three decision trees have branches which measure the effects of early or late collar removal with or without a cervical spine MRI. Divisions within a branch are the possibility of occurrence or nonoccurrence of complications resulting from the components of cervical spine management. Chance nodes are placed where the branches divide and are assigned probability values based on estimates from the literature.

Probabilities for chance events are found in the literature using the PubMed database of the National Library of Medicine and text accessible with Google Scholar. Peer reviewed medical reports and Web documents written by a medical professional in English published between 1998 and 2010 were reviewed for key data. No randomized control trial was identified which considered the outcomes in this analysis. Data was obtained for the most part from retrospective observational case series. Due to the absence of evidentiary data, sensitivity analysis subsequently described measured the outcomes over varying ranges of model inputs.

The sequence of modeled clinical events recorded at the end of a branch of the decision tree is analogous to a clinical history. Each tree contains about 40 branches depicting 40 different combinations of clinical events occurring over the course of management. The complexities of these categories range from the simplest type of case without a complication to others with one or more complications.

### Cervical Spine Management Complications

Over the course of simulations, the number of cases that cross a given branch depends on the exposure to events inherent to a cervical spine management strategy and the probabilities of these events. Table 1 shows the probability break down by delirium, increased intracranial pressure and aspiration or ventilator associated pneumonia possibly resulting from collar use [8]. Cell entries are ordered sequences of numbers corresponding to unstable, high risk and stable categories of TBI. The values are marginal probabilities, the difference in probability occurrences of late collar removal and early collar removal. Table 2 shows the probability break down of complications associated with transport to a MRI facility, increased intracranial pressure and aspiration resulting in ventilator associated pneumonia by early and late collar use [13], [14]. Cell entries are probabilities of occurrence of the complications for unstable, high risk and stable categories of TBI.

**Table 1:** Collar complications expressed as marginal probability*in percent according to categories of TBI.

Collar Complications	US/HR/S
Delirium	22/22/22
IICP	36/0/0
VAP	14/14/14

*Difference in occurrences of late and early collar removal.

US for Unstable, HR for High Risk and S for Stable

IICP is increased intracranial pressure

VAP is ventilator associated pneumonia

**Table 2:** MRI complications, early and late occurrence, expressed in percent probability according to categories of TBI.

MRI Complications	Early	Late
	US/HR/S	US/HR/S
Transport	14/14/9	14/14/9
IICP	72/0/0	0/0/0
VAP	18/18/18	18/18/18

US for Unstable, HR for High Risk and S for Stable

IICP is increased intracranial pressure

VAP is ventilator associated pneumonia

### Clinical Outcomes of Brain Injury

Primary, secondary brain injury and any additional harm to the brain resulting from cervical spine management are assessed for each branch of the decision tree to determine a health outcome. This assessment is a value corresponding to a score on a brain injury scale. No brain injury corresponds to zero on the scale, while greater degrees of brain injury are given higher values. The scale is based on survivability of brain injury, matching estimates of functional survival reported in the literature for high risk, unstable, and stable TBI. Functional survivals are assigned to values on the scale of less than 0.56; severe brain injury to values between 0.56 and 0.65; and death to values greater than 0.65. Thresholds are found through experimentation separating health outcomes according to their matching of frequencies of reported literature outcomes.

Since the measurement of clinical severity of brain injury is incomplete, distributions are used to express the uncertainties in severity. Sampling of distributions simulates the clinical vagaries in severity assessment for these comatose individuals. Gamma probability distributions are very flexible and are useful in describing heterogeneous observations such as observed in reports of brain injuries. The shape and scale parameters of gamma probability distributions define the locations and spread along the brain injury scale for primary and secondary brain injuries, as well as injury from cervical spine management. Gamma distributions are used to define the severity of primary and secondary brain injuries in the unstable, high risk and stable categories of severe TBI. For the unstable category of severe TBI the means for primary and secondary brain injury are 0.5000 and 0.0750, respectively. For the high risk category the means for primary and secondary brain injury are 0.7000 and 0.0200, respectively. For the stable category the means for primary and secondary brain injury are 0.5000 and 0.0200, respectively. The stable and unstable categories are set apart by the mean value of their secondary brain injury, while the stable and high risk categories are distinguished by the mean value of their of primary brain injury.

Brain injuries from increased intracranial pressure, ventilator associated pneumonia and delirium, as well as from potential complications during transport to a MRI facility and during the procedure itself are represented as a gamma distribution with mean of 0.025. Values for events resulting in brain injury are obtained by sampling their respective gamma probability distributions using TreeAge Pro. These results become part of the case's history. The total amount of brain injury is calculated by summing these elements of injury.

A case's health outcome based on brain injury is determined by the position of the total brain injury value on the relative brain injury scale. This scale is divided into regions related to the health outcomes. These regions are considered functional survival, severe brain injury and death as described above.

### Cervical spine instability and Quadriplegia

The presence or absence of cervical spine instability is a chance node on branches in each of the 3 trees. The risk of quadriplegia for a given cervical spine management strategy depends on its efficacy in reducing progression. Protection from quadriplegia for early collar removal is 0%; for late collar removal 80%; and for early and late collar removal with MRI 100%. These values are determined by expert opinion. The presence or absence of quadriplegia becomes part of the case's history. Quadriplegia is one of the four possible health outcomes along with functional survival, severe brain injury and death. Cervical spine instability assumes a baseline probability value of 2.5% [2] and, for sensitivity analysis varied up to 5.0%.

### Utilities of Health Outcomes

Each of the four health outcomes is assigned Quality Adjusted Life Years (QALYs) based upon its life expectancy and a utility factor. QALYs are common units of effect derived from different health outcomes and are used to measure the output of cervical spine management strategies. In order to standardize life expectancy for health outcomes, a case is assumed to be 40 years old. Life expectancy estimates from the literature in years for functional survival is 39.5 [6], for quadriplegia 20.0 [15] and for severe brain injury 20.0 [16]. Utilities of the modeled health outcomes are assessed based on the quality of overall well-being. No utility study is identified paralleling severe traumatic brain injury as presented in this study. However, health states of quadriplegia and brain injury states related to vascular disease are reported [17], [18]. Utility values corresponding to the modeled outcomes are functional survival 0.9, to quadriplegia 0.2, severe brain disability 0.1 and death 0.0. When quadriplegia occurs, the utility assigned to the case is the lesser of the two values. For example, quadriplegia with functional survival is assigned a value of 0.2; quadriplegia with severe brain disability is assigned a value of 0.1. The product of the life expectancy and utility value results in the QALY for a given case. The total QALY output of a management strategy is obtained by averaging the QALY for all cases.

### Description of a Case

**Figure 1:**
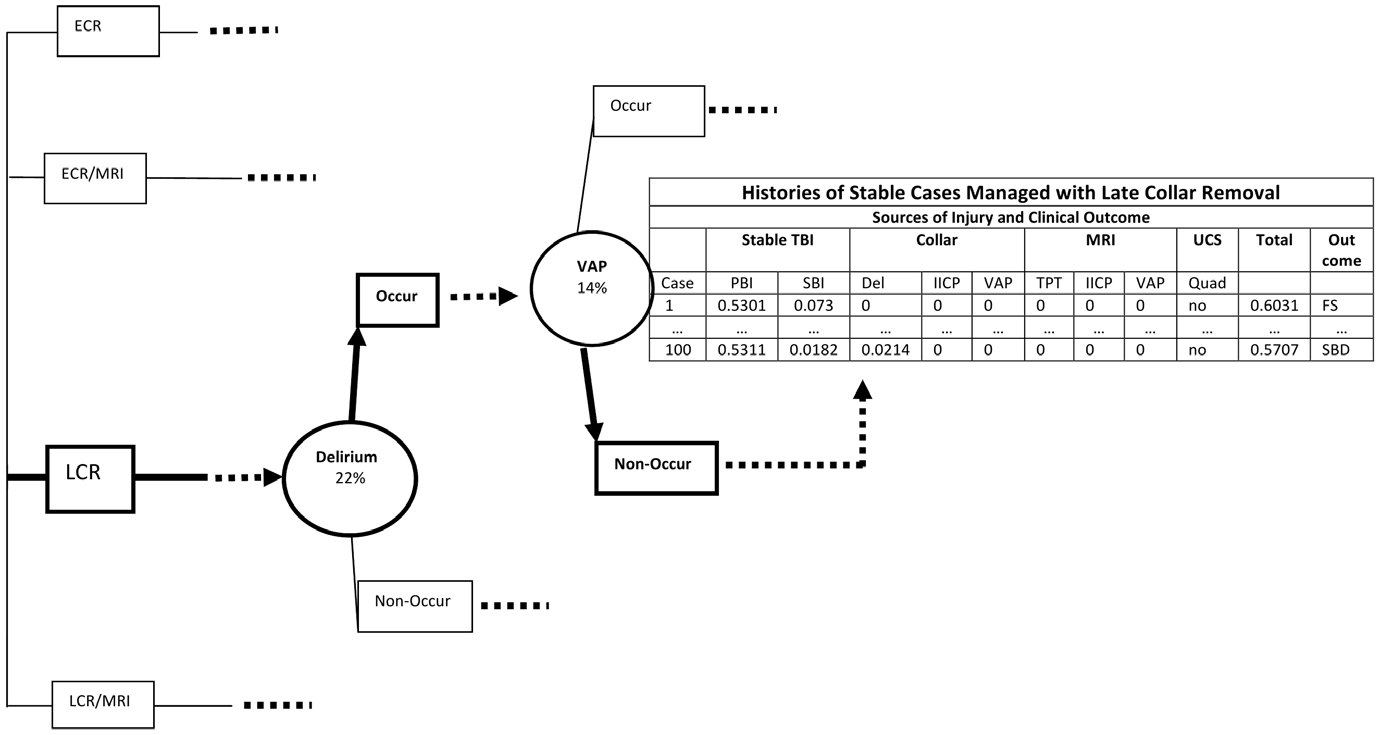
History of Events Leading to Severe Brain Disability Managed with Late Collar Removal for Case #100.

An example of a stable TBI case managed with late collar removal is illustrated in Figure 1. The bolding denotes the progression of a given case. In the history table in the figure, this case is identified as 100 and experienced an amount of primary brain injury of 0.5311 and secondary brain injury of 0.0182. These values are obtained by sampling the gamma distributions for primary and secondary brain injury.

At the delirium chance node, case 100 risks delirium with a probability of 22% and does experience delirium with additional brain injury of 0.0214. Furthermore, the uncertainty of developing ventilator associated pneumonia is depicted as a chance node. Progressing along the bolded pathway, one sees that aspiration pneumonia did not occur and review of the history table shows no brain injury resulting from aspiration pneumonia. Although the quadriplegia chance node is not illustrated, no quadriplegia occurs. At the end of case 100's clinical course, total brain injury is computed by summing primary, secondary and delirium which amounts to 0.5707. In the last column one sees that case 100's clinical outcome is that of severe brain disability.

### Sensitivity Analysis

The influence of cervical spine instability is studied with one-way sensitivity analysis with inputs of 2.5% and 5.0% used for probabilities of cervical spine instability while holding other inputs fixed. Tables 4 and 5 show selected results from these analyses. An idealized strategy with no quadriplegia and no cervical spine management brain injury is termed the benchmark and is used for comparison.

**Figure 2:**
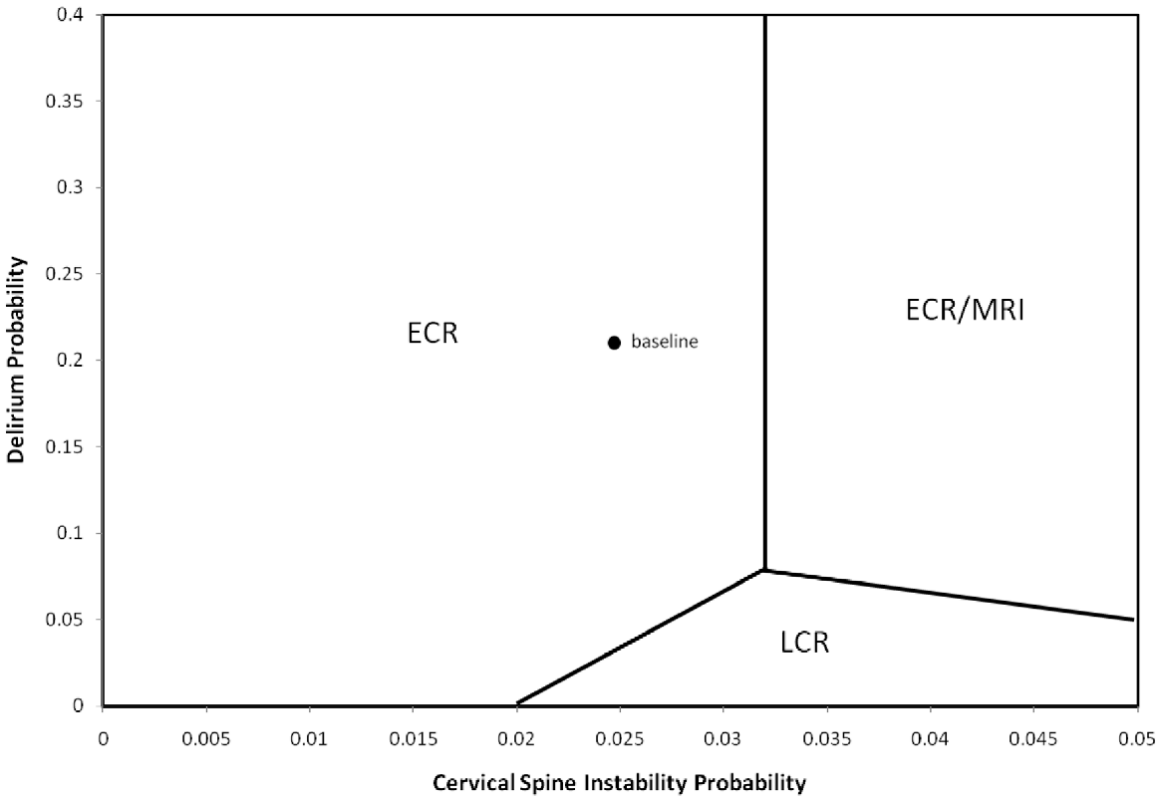
Two-Way Sensitivity Analysis, Delirium Probability vs. Cervical Spine Instability Probability in the Stable category of TBI.

**Figure 3:**
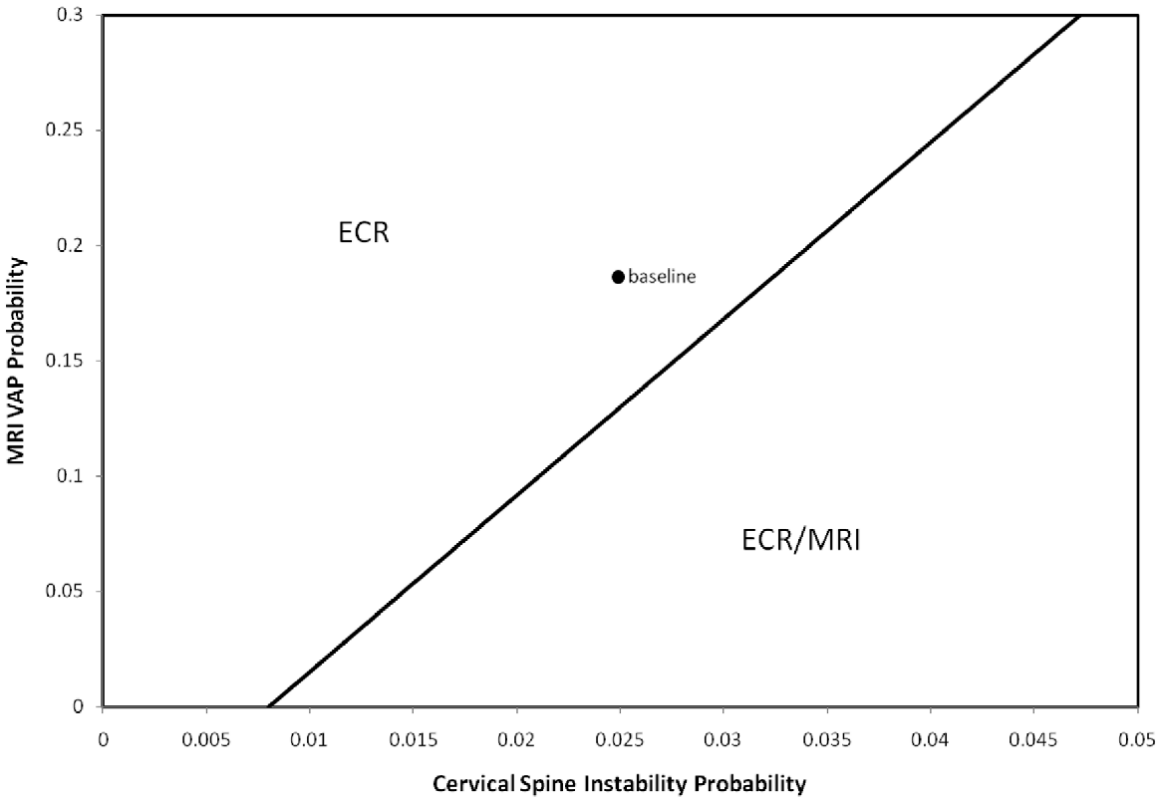
Two-Way Sensitivity Analysis, MRI VAP Probability vs. Cervical Spine Instability Probability in the Stable category of TBI.

Two-way sensitivity analysis is used to study the influence of varying probabilities on the rankings of cervical spine management strategies based on QALY results while other input values are held constant. For Figure 2 and Figure 3 the fixed input values are selected from Tables 1 and 2. In Figure 2, the probabilities of cervical spine instability and probabilities of delirium are varied. In Figure 3, the probabilities of cervical spine instability and aspiration pneumonia due to the MRI procedure are varied. Ranges of values for the probability of events define an area of interest. Coordinates of probability values serve as input for the model while holding other values fixed. For each coordinate the model permits the ranking of the management strategies based on QALY values. The process of selecting probability coordinates and assessing cervical spine management strategies is systematically repeated to define regions where superiority of a given strategy exists based on QALY value.

## Results

Table 3 shows occurrences when the base input values are used which includes a 2.5% probability of cervical spine instability. The layout of the table is organized into rows of clinical outcomes and QALYs. The columns contain the four cervical spine management strategies as well as an idealized benchmark. Strategies and benchmark are further divided into the three TBI categories, unstable, high risk and stable. Results are summarized based on 1,000 cases. Early collar removal has the highest number of functional survivals and quadriplegics among the categories of TBI. With respect to severe brain disabilities, early collar removal results show a mixed pattern among the categories when compared to other strategies. Early collar removal has lower counts of severe brain disabilities among the unstable and stable categories but a higher count among the high risk category when compared to other cervical spine management strategies. Early collar removal has the fewest count of deaths and most QALYs among the categories when compared to other strategies.

**Table 3:** Expected Clinical Outcomes per 1,000 and QALYs By CSM Strategy and Patient Category with 2.5% Probability of Cervical Spine Instability.

Clinical Outcome*	Benchmark			LCR			LCR/MRI			ECR/MRI			ECR		
	US	HR	S	US	HR	S	US	HR	S	US	HR	S	US	HR	S
FS	394	165	610	349	151	586	331	140	572	330	153	594	384	161	595
Quad	0	0	0	2	1	3	0	0	0	0	0	0	10	4	15
SBD	216	167	175	218	162	182	218	157	186	218	162	179	216	167	175
Dead	390	668	214	431	687	229	451	703	241	451	685	226	390	668	214
QALYs	14.51	6.17	22.20	12.87	5.71	21.23	12.22	5.30	20.73	12.20	5.76	21.50	14.30	6.08	21.59

FS is Functional Survival

Quad is Quadriplegic

SBD is Severe Brain Disability

QALYs are Quality Adjusted Life Years

US for Unstable, HR for High Risk and S for Stable

LCR is Late Collar Removal

ECR is Early Collar Removal

*Totals of clinical outcomes may vary by one due to rounding

The benchmark has the highest number of functional survivals among categories of TBI and has no quadriplegics. With respect to severe brain disabilities and death, the benchmark has the same counts as early collar removal. The benchmark has the most QALYs when compared to any strategy.

The benchmark has greater numbers of cases with more favorable clinical outcomes than the clinical strategies. The additional brain injury from complications of the four cervical spine management strategies increases total brain injury thus resulting in greater numbers of cases with less desirable clinical outcomes.

Table 4 has three sections of QALY results broken down by unstable, high risk and stable categories of TBI and calculated with a 2.5% probability of cervical spine instability. The layout of each section is similar. In the section's rows are the benchmark and the four cervical spine management strategies. The section's columns are the rank ordering of strategies, net QALYs, lost QALYs to quadriplegia, lost QALYs to brain injury resulting from cervical spine management and total lost QALYs. Table 5 has a similar layout to Table 4 and shows QALY results for the stable category of TBI calculated with 5.0% probability of cervical spine instability.

**Table 4:** QALYs Resulting from Quadriplegia and CS Management Complications with a 2.5% Probability of Cervical Spine Instability in the categories of TBI.

Unstable					
	Rank	Net QALYs	Lost QALYs to Quad	Lost QALYs to CSM BI	Total Lost QALYs
Benchmark	NA	14.51	0	0	0
ECR	1	14.30	0.21	0	0.21
ECR/MRI	4	12.14	0	2.37	2.37
LCR/MRI	3	12.16	0	2.35	2.35
LCR	2	12.79	0.03	1.69	1.72

High Risk					
	Rank	Net QALYs	Lost QALYs to Quad	Lost QALYs to CSM BI	Total Lost QALYs
Benchmark	NA	6.17	0	0	0
ECR	1	6.08	0.09	0	0.09
ECR/MRI	2	5.76	0	0.41	0.41
LCR/MRI	4	5.30	0	0.87	0.87
LCR	3	5.71	0.02	0.44	0.46

Stable					
	Rank	Net QALYs	Lost QALYs to Quad	Lost QALYs to CSM BI	Total Lost QALYs
Benchmark	NA	22.20	0	0	0
ECR	1	21.59	0.61	0	0.61
ECR/MRI	2	21.50	0	0.70	0.70
LCR/MRI	4	20.73	0	1.47	1.47
LCR	3	21.23	0.11	0.86	0.97

TBI is Traumatic Brain Injury

QALYs are Quality Adjusted Life Years

LCR is Late Collar Removal

ECR is Early Collar Removal

Quad is Quadriplegia

CSM BI is Cervical Spine Management Brain Injury

NA is not applicable

**Table 5:** QALYs Resulting from Quadriplegia and CS Management Complications with a 5.0% Probability of Cervical Spine Instability in the Stable category of TBI.

Stable					
	Rank	Net QALYs	Lost QALY to Quad	Lost QALYs to CSM BI	Total Lost QALYs
Benchmark	NA	22.20	0	0	0
ECR	3	21.30	0.90	0	0.90
ECR/MRI	1	21.69	0	0.51	0.51
LCR/MRI	4	20.91	0	1.29	1.29
LCR	2	21.34	0.17	0.69	0.86

TBI is Traumatic Brain Injury

QALYs are Quality Adjusted Life Years

LCR is Late Collar Removal

ECR is Early Collar Removal

Quad is Quadriplegia

CSM BI is Cervical Spine Management Brain Injury

NA is not applicable

In Table 4 for the unstable category of TBI, the ranking of strategies based on net QALY values in descending order is ECR (14.3), LCR (12.79), LCR/MRI (12.16) and ECR/MRI (12.14). For the high risk category, the ranking of strategies based on net QALY values in descending order is ECR (6.08), ECR/MRI (5.76), LCR (5.71) and LCR/MRI (5.30). The ranking for the stable category is ECR (21.59), ECR/MRI (21.50), LCR (21.23), and LCR/MRI (20.73).

In Table 5 for the stable category of TBI with a 5% probability of unstable cervical spine, the ranking of strategies based on net QALY values in descending order is ECR/MRI (21.69), LCR (21.34), ECR (21.30) and LCR/MRI (20.91).

In Tables 4 and 5 the benchmark shows no loss of QALYs from quadriplegia and brain injury from cervical spine management. This finding is consistent with the definition of benchmark, being 100% efficacious in preventing quadriplegia without causing brain injury from cervical spine management. All MRI based strategies show no loss of QALYs from quadriplegia consistent with the assumption that MRI detects all cases of cervical spine instability.

A two-way sensitivity analysis varying the probabilities of cervical spine instability and collar delirium for the stable category of TBI is displayed in Figure 2. The y-axis shows the probability of delirium varying from 0 to 0.4. The x-axis shows the probability of cervical spine instability varying from 0 to 0.05. The labels of these regions, ECR, LCR and ECR/MRI, are the highest ranked of the four strategies when ordered by net QALYs. A region's label defines a combination of inputs for which a strategy is superior and thus a preferred approach. Lines separating regions indicate equivalence of QALYs and indifference in choice between adjacent strategies. The point labeled baseline is in the ECR region. These base inputs correspond to the values 0.025 for cervical spine instability and 0.22 for collar delirium. At a low probability of cervical spine instability, ECR is superior regardless of the possibility of collar delirium. At higher probabilities of cervical spine instability the preference for ECR is replaced by a choice between LCR or ECR/MRI. The probability of delirium now controls the choice. At low probability of delirium LCR is preferred and at higher probability of delirium ECR/MRI is favored.

A two-way sensitivity analysis varying the probabilities of cervical spine instability and ventilator associated pneumonia due to MRI in the TBI category of stable cases is displayed in Figure 3. The y-axis shows the probability of ventilator associated pneumonia varying from 0 to 0.3. The x-axis shows the probability of cervical spine instability varying from 0 to 0.05. There are two regions one labeled ECR and the other ECR/MRI. The labeling scheme for regions is the same as in Figure 2. The point labeled baseline is in the ECR region. These base inputs correspond to the values 0.025 for cervical spine instability and 0.18 for ventilator associated pneumonia due to the MRI procedure. At lower probabilities of aspiration or ventilator associated pneumonia due to the MRI procedure; ECR/MRI is more favored.

Tables 4 and 5 show how the increase in the probability of cervical spine instability from 2.5% to 5.0% results in a change in strategy preference. The balance of losses in QALYs from quadriplegia and cervical spine management brain injury favor ECR at a probability of 2.5% cervical spine instability but changes to ECR/MRI when the probability increases to 5.0%. This change in strategy is present only in the stable category of TBI and does not occur in the high risk and unstable categories.

## Discussion

The major goal of cervical spine management should be to maximize functional survival from brain injury while minimizing quadriplegia. There is difficulty judging the success of this objective merely by counting numbers of clinical outcomes. As an example, in Table 3, compare the results of ECR/MRI with ECR within the stable category of TBI. ECR/MRI has one less case of functional survival than ECR and eliminates the 15 cases of quadriplegia. However, there are four more cases of severe brain disability and 12 more deaths. There is a trade-off of 15 cases of quadriplegia and one functional survival in exchange for 12 deaths and four cases of severe brain disability.

Which of these two cervical spine management strategies is preferable? QALYs provide an answer by converting clinical outcomes to a common unit for comparison. Accordingly, clinical strategies can be compared using QALYs. In the previous example, the QALYs for ECR are 21.59 which exceed the 21.50 QALYs for ECR/MRI suggesting that ECR is favored. Given the tight spread of QALYs, one can appreciate the reason for the divergence of opinion that exists regarding the selection of a strategy.

While there has been progress in understanding these types of injuries, a great deal still needs to be learned to improve health outcomes [19]. Therapies and diagnostic procedures for TBI have improved outcomes over time [20]. Cervical collar use has been a common treatment for severe TBI with the intent of preventing possible progression of a masked neck injury [21]. Until recently clinical reports have not contained information about MRI, an imaging service only now available in many health care settings. In the setting of brain injury there is little information assessing the influence of MRI or MRI combined with collar usage on the clinical outcomes of functional survival, severe brain injury, and quadriplegia. With the observation in this report, unstable, high risk and stable categories may benefit from different management strategies.

The conceptual premises presented are developed from a detailed review of pertinent studies over 25 years and include management complications specific to TBI; such as increased intracranial pressure and complications that anyone critically ill might experience [2]. These complications include ventilator associated pneumonia and possible hypotensive episodes during transport to a MRI. These and other complications from transport within a facility have been understudied as indicated by the Agency for Healthcare Research and Quality [11]. Advancements in technology and quality improvements in health care have reduced the numbers of complications occurring over time. Literature definitions of ventilator associated pneumonia have become more precise over the past 25 years. Antibiotics play a direct role in reducing direct mortality from pneumonias [22]. Reported complications of increased intracranial pressure also reflect trends in improved management over this time frame. Early and more precise monitoring of increased intracranial pressure is at the forefront of severe TBI management and has changed the numbers of reported complications from this event [23].

Using publications as a source of information about this unusual variant of severe TBI reveals two significant difficulties. First there are no controlled clinical trials investigating these injures. Thus, there is no longitudinal data of follow up from the time of trauma, through recovery, rehabilitation and long term survival. In lieu of test and control arms of a clinical trial, snapshots of recovery, rehabilitation and life expectancy are assessed through diverse cohort and case study reports. Thus, the overall conclusions regarding clinical outcomes are weak and professional organizations are hesitant to commit to clinical practice guidelines without solid data or studies.

The second difficulty is the inconsistency in terms used to describe aspects of brain injury and its care. All the specialists involved in the management of this type of injury may use different terms specific to their discipline to describe features of TBI. The absence of a universal reporting standard is a key barrier to the sharing and reuse of published clinical data from within these medical disciplines.

A controlled and structured vocabulary defining clinical concepts and relations for brain trauma, developed through interdisciplinary consensus would increase the value of these publications. A clinical ontology for TBI in the era of the electronic medical record would facilitate the collection and pooling of data from multiple heath care facilities across various medical professions and would accelerate the understanding of cervical spine management strategies. In the absence of controlled clinical studies as well as consensus guidelines for this dilemma, the development of such an ontology would appear as a priority.

In clinical practice, the decision to select a cervical spine management strategy is based on the trade-off between minimizing incremental brain injury and reducing the risk of quadriplegia. Despite the infrequency of cervical spine instability in cases with a negative cervical spine exam and negative cervical spine CT, a cervical collar or cervical MRI is often dogmatically ordered. Perhaps the reasons for these orders are due to simplicity of use, availability or incomplete understanding of their drawbacks.

In hindsight, the decision not to use a collar or MRI in a case progressing to quadriplegia could be considered as negligence in care. Incremental brain injury resulting from late collar removal and cervical spine MRI is probably not considered in such a judgment. Complications of cervical spine management and their contributions to additional brain injury may be overlooked in accounting for less desirable clinical outcomes. Coma and the underlying high mortality associated with severe TBI mask these features.

Two aspects of the uncertainty in this problem are modeled. The first is mimicking the occurrence of complications and the second is the expression of the incomplete understanding of brain injury severity. In imitating the occurrence of complications, chance nodes within the decision tree are used to manipulate the probabilities by use of the Monte Carlo process. The occurrence of ventilator associated pneumonia, delirium, increased intracranial pressure, cervical spine instability and quadriplegia are thus simulated.

Distributions are used to express the incomplete understanding of brain injury in terms of clinical severity. Samplings of these distributions imitate the clinical sense of severity arising from primary and secondary brain injury sources and complications of cervical spine management. The relative severity of brain injury from each source is determined by the locality of the source's values on the brain injury scale. Ultimately, the amounts of brain injury can determine a clinical outcome. Each individual's severity of injury is chosen from a range of possible values, while point estimates of probabilities and utilities are common to all individuals. The fixed nature of point estimates lends itself to one-way and two-way sensitivity analysis, which is helpful in identifying the effect of variation of values in selected variables. A drawback of this approach in a model with many variables is the assumption that the non-participating variables are valid and unchanging. An alternative approach to sensitivity analysis is the use of a distribution instead of point estimates. Variables which were constant now change values with periodic sampling of the incorporated distributions. This technique, called probability sensitivity analysis, permits an integrated assessment of the underlying uncertainties. However, the appreciation of the influence of a specific variable on outcomes is diminished.

In assessing the validity of the model, the following points should be considered. The model permits the occurrence of up to six chance complications without altering strategy thus simulating an extreme situation. No supporting literature is found describing the tolerances of clinicians for staying a course of therapy in the face of mounting complications. However, four or more complications for a case are uncommon in the model and the influence of accumulating brain injuries is lessened by not modeling complications as a direct cause of death.

Limitations in this model include the omission of awaking from coma. One might argue that late collar removal is a “wait and see” strategy delaying a decision until awakening. After awakening, clinical assessment would guide subsequent management and reduce the numbers of cervical spine MRIs. The probability of awakening within two weeks is estimated at 0.5 [24], [25]. However, in many such cases cognitive abilities after awakening may be sufficiently impaired limiting awareness and responsiveness. Future studies may incorporate time to awakening as a random variable structured as a Markov process.

No clinical study, controlled or otherwise, measuring a cervical collar's efficacy in preventing quadriplegia is identified in the literature. A recent cadaver study suggests that collar use may be detrimental [26]. Findings show that cervical collar use increases the anatomic separation of segments in the neck at the site of ruptured ligaments. This concept imposes an opposite effect than intended and may contribute to cervical spine instability. For the model, expert opinion is used to estimate the ability of the cervical collar to protect against quadriplegia at 80%. This value may be overstated because first responders commonly apply an extrication collar in trauma with any suggestion of neck injury. Offsetting the optimism that the collar prevents quadriplegia is the assumption that all cases of cervical spine instability managed without a cervical collar or cervical MRI progress to quadriplegia.

Diagnosing cervical spine instability with damaged ligaments using MRI is highly accurate [27]. Hence the consequences of false information from a cervical spine MRI are not modeled. It is assumed that a positive cervical MRI is followed with successful surgical correction or cervical stabilization with a halo brace. The mortality directly related to these corrective procedures is reported to range between 0.4% and 2.5% [28], [29] and is not modeled.

The low occurrence of severe TBI with cervical spine instability due solely to ligament damage and the complexities of conducting a clinical trial for this injury explain in part why no registry is identified specific for this condition. Furthermore, a clinical trial evaluating the consequences of decisions in medical care during the early management phase would have lifetime of sequelae and would be quite costly. Due to the absence of an identified external data source, a comparison of modeled results to a test data set has not been conducted. This is an important step in a model's validation. Sensitivity analysis across factors and values is used as a comparison technique to identify patterns of data inputs to identify preferred cervical spine management strategies.

The model has a utilitarian perspective determining the greatest average health benefit for all cases over a lifetime. In part, this is achieved through a linear utility scale and QALYs but does not evaluate the resource cost of the four strategies. Study findings might differ if a cost-effectiveness evaluation is performed.

### Conclusion

Despite the acknowledged limitations of this model, sensitivity testing of variables over a wide range of relevancy shows results that are clinically plausible. The important aspects of this problem are presented carefully describing the information, rationale and assumptions. The analysis is constructed in the framework of three decision trees. The clinical logic and parts of cervical spine management are systematically altered to identify changes that might improve health outcomes. As in any model, the complexity of programming restricts the inclusion of all conceivable issues.

In the absence of controlled clinical studies, four cervical spine management strategies using available data are modeled as possible approaches to improve health outcomes in severe traumatic brain injury. Results support the use of early collar removal over a wide range of realistic inputs. There is a cautionary note in unconditionally using early collar removal for an individual without considering cervical spine instability and management complications. A probability of cervical spine instability greater than 2.5% or probability assessments of cervical spine management complications lower than baseline may favor either late collar removal or a strategy using a cervical spine MRI.
